# Strain-Hardening and High-Ductile Behavior of Alkali-Activated Slag-Based Composites with Added Zirconia Silica Fume

**DOI:** 10.3390/ma12213523

**Published:** 2019-10-27

**Authors:** Jeong-Il Choi, Se-Eon Park, Huy Hoàng Nguyễn, Sang Lyul Cha, Bang Yeon Lee

**Affiliations:** 1 Biohousing Research Center, Chonnam National University, 77 Yongbong-ro, Buk-gu, Gwangju 61186, Korea; himancji@naver.com; 2 School of Architecture, Chonnam National University, 77 Yongbong-ro, Buk-gu, Gwangju 61186, Korea; box6282@naver.com (S.-E.P.); hoanghuyjnu92@gmail.com (H.H.N.); 3 Department of Civil and Environmental Engineering, Korea Advanced Institute of Science and Technology, 291 Daehak-ro, Yuseong-gu, Daejeon 34141, Korea; maikuraki@kaist.ac.kr

**Keywords:** alkali-activator, composite, silica fume, slag, tensile strain capacity

## Abstract

This paper presents an experimental study on the effects of zirconia silica fume on the composite properties and cracking patterns of fiber-reinforced alkali-activated slag-based composites. Four mixtures were prepared with added zirconia silica fume and varying water-to-binder ratio. Polyethylene fiber was used as a reinforcing fiber for all the mixtures at a volumetric ratio of 2.0% cubic specimens and uniaxial tensile specimens were prepared to evaluate their density, compressive strength, and tensile behavior. The test results demonstrated that the compressive strength, tensile strength, and tensile strain capacity of the composite can be simultaneously improved by incorporating zirconia silica fume. A mixture incorporating zirconia silica fume showed high-ductile behavior of 26.5% higher tensile strength, and 13.7% higher tensile strain capacity than the mixture without zirconia silica fume. The composite with added zirconia silica fume also showed excellent cracking patterns, i.e., narrow crack spacing and crack width.

## 1. Introduction

Concrete is an economic construction material with relatively high strength and durability, and is the most frequently used construction material along with steel. Concrete’s performance can also be fairly easily adjusted by selecting its constituent materials, and by varying their proportion in the mixture. However, concrete also has low tensile strength and brittle behavior, which is an inherent disadvantage [1,2]. Cement production also generates large amounts of carbon dioxide. Previous studies have reported that about 1 ton of carbon dioxide is emitted for each ton of cement [3]. It has also been reported that about 5% of the world’s total artificially generated carbon dioxide is produced by the cement industry [4]. 

As a new construction material to mitigate the disadvantages of concrete, fiber-reinforced alkali-activated slag or geopolymer composite is environmentally friendly because it can be produced using industrial byproducts [5,6,7,8,9,10,11]. It also has high ductility, of over 3%, under a tensile load. In particular, unlike fly ash-based geopolymers, alkali-activated slag-based binding materials have been shown to develop strength even without high-temperature curing [12,13,14,15,16,17]. Previous studies have also reported that electric arc furnace slag can be used as a recycled aggregate [18,19,20]. They investigated experimentally the structural behavior of full-scale structural members realized with this kind of recycle aggregates as full replacement of natural aggregates. 

Lee et al. (2012), for example, experimentally demonstrated that alkali-activated slag-based composites, which were prepared using three combinations of activators and blast furnace slag as a binding material, reinforced with polyvinyl alcohol (PVA) fiber, had a compressive strength of 30.6 MPa and a maximum tensile strain capacity of 4.7% [8]. Choi et al. (2015) developed composites with a viscosity of 0.86 Pa s, a yield strength of 18 Pa, a compressive strength of 18.3 MPa, a tensile strength of 2.26 MPa, and a tensile strain capacity of 2.38% using an alkali-activated slag-based binding material [7]. They used a water-to-binder ratio of 40% and PVA fiber at a volumetric ratio of 1.3%. Choi et al. (2016a) experimentally demonstrated an ultra-high-ductile alkali-activated composite with a tensile strength of 13.1 MPa and a tensile strain capacity of 7.5% using an alkali-activated slag-based binding material and a polyethylene (PE) fiber [5]. Choi et al. (2016b) also compared the performance of a cement-based composite to an alkali-activated slag-based composite with the same water-to-binder ratio [6]. The experimental results showed that the alkali-activated slag-based composite had lower strength than the cement-based composite, but had a higher tensile strain capacity and a better cracking pattern (i.e., a smaller crack spacing and width). Lee et al. (2017) investigated the effect of a defoamer on the compressive strength and tensile strain capacity of a PE fiber-reinforced alkali-activated slag-based composite, and reported that mixing in a small amount of defoamer improved the compressive strength and tensile strain capacity of the composite [9]. Kwon et al. (2018) investigated the tensile behavior and cracking pattern of fiber-reinforced slag-based composites depending on fiber type (PP: polypropylene, PE: polyethylene, and PBO: polyparaphenylene-benzobisethiazole) and water-to-binder ratio [12]. The experimental results showed that the PE fiber-reinforced composite had the highest tensile strain capacity, while the PBO fiber-reinforced composite had the highest tensile strength and the smallest crack spacing and width. Nguyễn et al. (2018) evaluated the self-healing performance of slag-based composites prepared using calcium hydroxide as an activator, and showed that under the same conditions, slag-based composites more rapidly reduced the crack width but recovered strength to a lower level compared with the cement-based composites [14]. Nguyễn et al. (2019) investigated the self-healing performance of alkali-activated slag-based composites, prepared using calcium hydroxide, sodium hydroxide, and sodium silicate alone. They reported that the material prepared with calcium hydroxide as the activator had the highest self-healing performance and the best tensile behavior after self-healing [13]. 

Although alkali-activated slag-based composites have been actively studied, most previous studies have investigated the effect of the type of activator, the water-to-binder ratio, and the fiber types. By comparison, very few have investigated the effects of admixtures on the properties of alkali-activated slag-based composites. Among them, silica fume is one of more widely used supplementary materials for increasing concrete strength. That strengthening is due to the additive’s ultra-fine particle size and pozzolanic reaction, which fills the voids between larger particles and produces secondary hydrates by chemical reaction with lime [21,22,23]. Silica fume also improves durability by densifying the microstructure of the concrete [24]. 

To improve the performance of alkali-activated slag-based composites, the present study investigated the effect of silica fume on their mechanical performance. 

## 2. Materials and Methods 

### 2.1. Materials and Mixture Proportion

Ground granulated blast furnace slag (GGBS) was employed as the source material. The Blaine fineness and the specific gravity of the GGBS were 4320 cm^2^/g and 2.92, respectively. Table 1 shows the chemical compositions of the GGBS. Zirconia silica fume (ZSF) containing a small amount of zirconium was used as a supplementary material. Table 1 shows the chemical compositions of ZSF. The specific surface area of ZSF, measured by the Brunauer, Emmett, and Teller (BET) method of adsorption of nitrogen gas, was 7.05 m^2^/g. The chemical compositions of the GGBS and ZSF were measured by the X-ray fluorescence (XRF) technique (Model: Axios Minerals). Calcium hydroxide and sodium sulfate were used as the activators of the binders, and mixed in the form of powder to prevent quick setting. 

**Figure 1:**
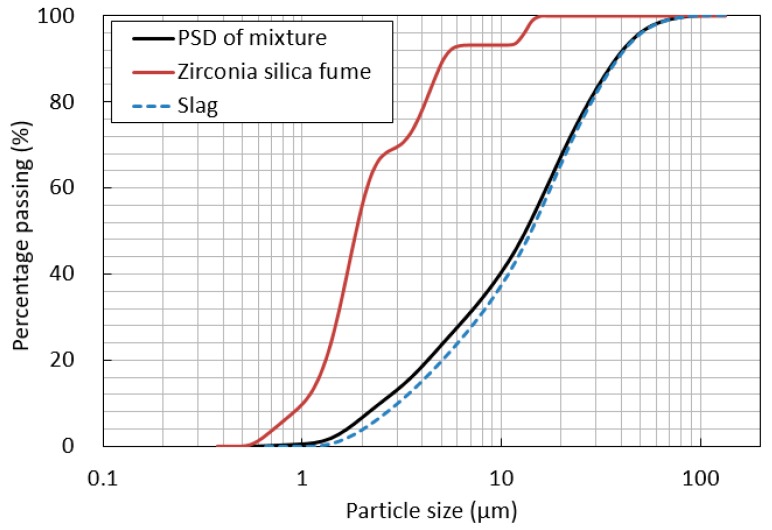
Particle size distribution.

Figure 1 shows the particle size distribution (PSD) of the GGBS, ZSF, and a mixture of GGBS and ZSF, in which 5% of the total binder was replaced with ZSF. It was measured using a Beckman Coulter LS230 laser diffraction particle size analyzer (Indianapolis, IN, USA). The particle size distribution of the GGBS was very different from that of ZSF. However, the particle size distribution of the GGBS and ZSF mixture was not significantly different from that of the GGBS, because the replacement ratio of GGBS with ZSF was only 5%. A high-range water-reducing agent (HRWRA), a polycarboxylate-based superplasticizer, was used to control the viscosity of the paste to ensure uniform distribution of the fiber. A defoamer, a mixture of surface active agents and mineral substances without silicone, was used to prevent the unintended generation of foams in the specimen during the manufacturing process. The PE fiber used as a reinforcing fiber had a length of 18 mm and a diameter of 12 μm, therefore the aspect ratio of the PE fiber was 1500. The tensile strength, elastic modulus and density of the PE fiber were 2700 MPa, 88 GPa, and 0.97 g/cm^3^, respectively.

Table 2 shows the mixture proportions used in the present study. The mixtures were designed to investigate how ZSF affected the composite properties of the alkali-activated slag-based composite. In all the mixtures, the weight ratios of calcium hydroxide and sodium sulfate were respectively 8.4% and 3.4% of the weight of the GGBS. The reference mixture was W21-S0, which had a water-binding material ratio of 21%. The W21-S5 mixture was prepared by replacing 5% of the binding materials of the reference mixture with ZSF. The W25-S5 and W30-S5 mixtures were prepared to investigate the effect of water-to-binder ratio on mixtures with ZSF.

### 2.2. Specimen Preparation 

To prepare the specimens, the GGBS, activators and ZSF, which were powder type materials, were dry-mixed for three min in a planetary mixer. Then, a HRWRA and a defoamer were added with water. The fiber was sequentially mixed in after checking that the paste was homogeneous and had proper viscosity, to ensure the fiber would be homogeneously dispersed. After adding the fiber, the mixtures were mixed for 5 min until the fiber was homogenously dispersed. Following the mixing process, three 50 mm cube specimens were prepared from each mixture, for compressive strength and density measurements. 

Following the Japan Society of Civil Engineers (JSCE) recommendation [25], five dumbbell-shaped specimens were prepared with each mixture to conduct the uniaxial tension tests. The gauge length of the tension test specimens was 80 mm, and the cross-sectional area within the gauge length was constant at 30 mm × 13 mm. After preparing the specimens, the specimens were covered with a plastic sheet to prevent water evaporation and cured at room temperature for two days. Then, the specimens were demolded and cured in a curing-water tank at 23 °C ± 3 °C to the age of 28 days.

### 2.3. Test Methods

The unintentional generation of large pores during the specimen preparation process can affect the mechanical properties of the resulting specimens. This possibility was addressed by measuring the density of the specimens. To calculate the density of individual specimens, the weight of the specimen in air and in water were measured and then the density was calculated using Equation (1) [26].

(1) $$\rho =\frac{W_{A}}{W_{A}-W_{W}}\times \rho_{w},$$

where $W_{A}$ is the weight of specimen in air, $W_{W}$ is the weight of specimen in water, $\rho_{w}$ is the density of water. 

The compressive strength of specimens from each mixture was measured according to ASTM C109 [27]. The tension test was performed using an electric tension test machine according to the JSCE recommendation [25]. The tensile load was applied by controlled displacement at a rate of 0.1 mm/min, which corresponds to a quasi-static load at a strain rate of 2.08 × 10^−5^ 1/s [28,29]. The load was measured using a load-cell with a capacity of 20 kN, which was attached to the machine. The deformation within a gauge length of each specimen was measured using two linear variable differential transducers, which were attached to both sides of each specimen. 

To investigate cracking patterns, the number of cracks occurring in the gauge length (80 mm) of the specimens was measured using a magnifier after the tension test. The crack spacing was calculated by dividing the number of cracks by the gauge length. It was assumed that all deformation occurred at cracks, because without cracks deformation of the matrix was much smaller than the crack width. For this assumption, the average crack width was calculated by dividing the total deformation in the gauge length when the specimens reached the maximum tensile strength, by the number of cracks.

Scanning electron microscopic (SEM, JEOL, Tokyo, Japan) and energy dispersive X-Ray spectroscopy (EDS) were used to investigate the morphology and chemical compositions of composites. The accelerating voltage and the probe current of the SEM and EDS configuration were 20 kV and 71.5 nA, respectively. It is necessary to mention that the SEM/EDS investigation was only performed using the secondary electron imaging mode.

## 3. Results and Discussion

### 3.1. Density 

**Figure 2:**
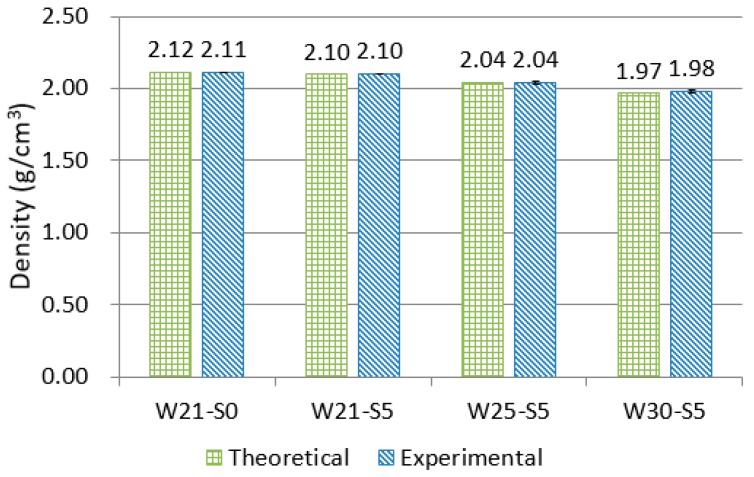
Theoretical and experimental density of mixtures.

Figure 2 shows the measured density and the theoretical density of the mixtures. The theoretical density was calculated using the density of the component materials and their proportion in each mixture. The difference in density was determined to be less than 0.5%, which indicated that unintended pores were not generated during the specimen’s manufacture or curing. This confirmed that the properties of the composites were not affected by unintentional large pores.

### 3.2. Compressive Strength 

**Figure 3:**
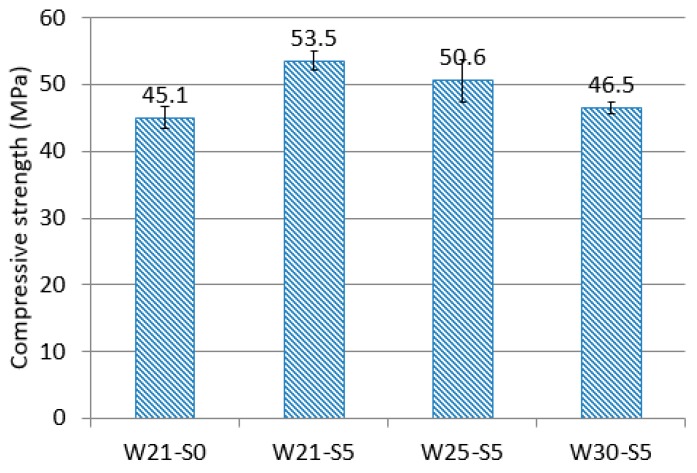
Average compressive strength of three cubic specimens.

Figure 3 shows the compressive strength of each mixture. The maximum coefficient of variation was 6.2%. Replacing 5% of the total amount of the binding materials with ZSF increased the compressive strength by 18.8%. This may be because ultra-fine ZSF particles undergo the pozzolanic reaction, and increase packing density, making the microstructure denser. In the mixtures that included ZSF, strength declined as the water-to-binder ratio was increased. The compressive strength of the W30-S5 mixture, prepared by replacing 5% of the total amount of the binding materials with ZSF, and with a water-to-binder ratio of 30%, was similar to that of the reference mixture.

### 3.3. Uniaxial Tensile Behavior

**Figure 4:**
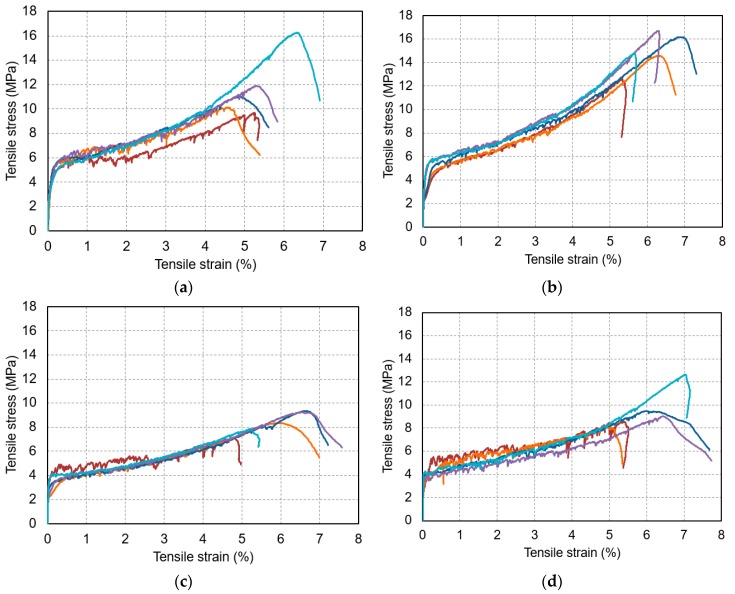
Tensile stress and strain curves: (**a**) W21-S0, (**b**) W21-S5, (**c**) W25-S5, and (**d**) W30-S5.

Figure 4 shows the tensile stress and strain curves of all the specimens. All four mixtures showed strain-hardening behavior. When strain was increased after the first crack was generated, stress significantly increased. A previous study showed that stress was greatly decreased by the occurrence of cracks when a hydrophobic PE fiber was used [30]. However, there was no significant decrease in stress in the mixtures used in the present study when a crack was generated.

**Figure 5:**
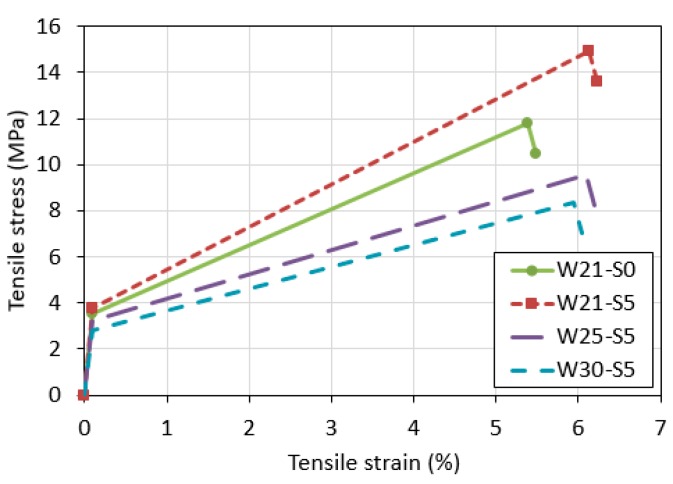
Comparison of tensile stress and strain curves of all mixtures.

Based on the fact that there was little decrease in stress, it can be inferred that the interfacial bond between the fiber and the matrix was high. Figure 5 shows the average tensile stress and strain curves of each mixture, which are expressed using a trilinear model. The W21-S5 mixture, which was prepared by replacing 5% of the binding materials of the reference mixture (W21-S0), exhibited a tensile strength and a tensile strain capacity that were much higher than the reference mixture. The experimental results indicated that the substitution of ZSF can improve both tensile strength and tensile strain capacity. In the mixtures prepared using 5% ZSF substitution, the tensile strength and the tensile strain capacity decreased as the water-to-binder ratio was increased.

**Figure 6:**
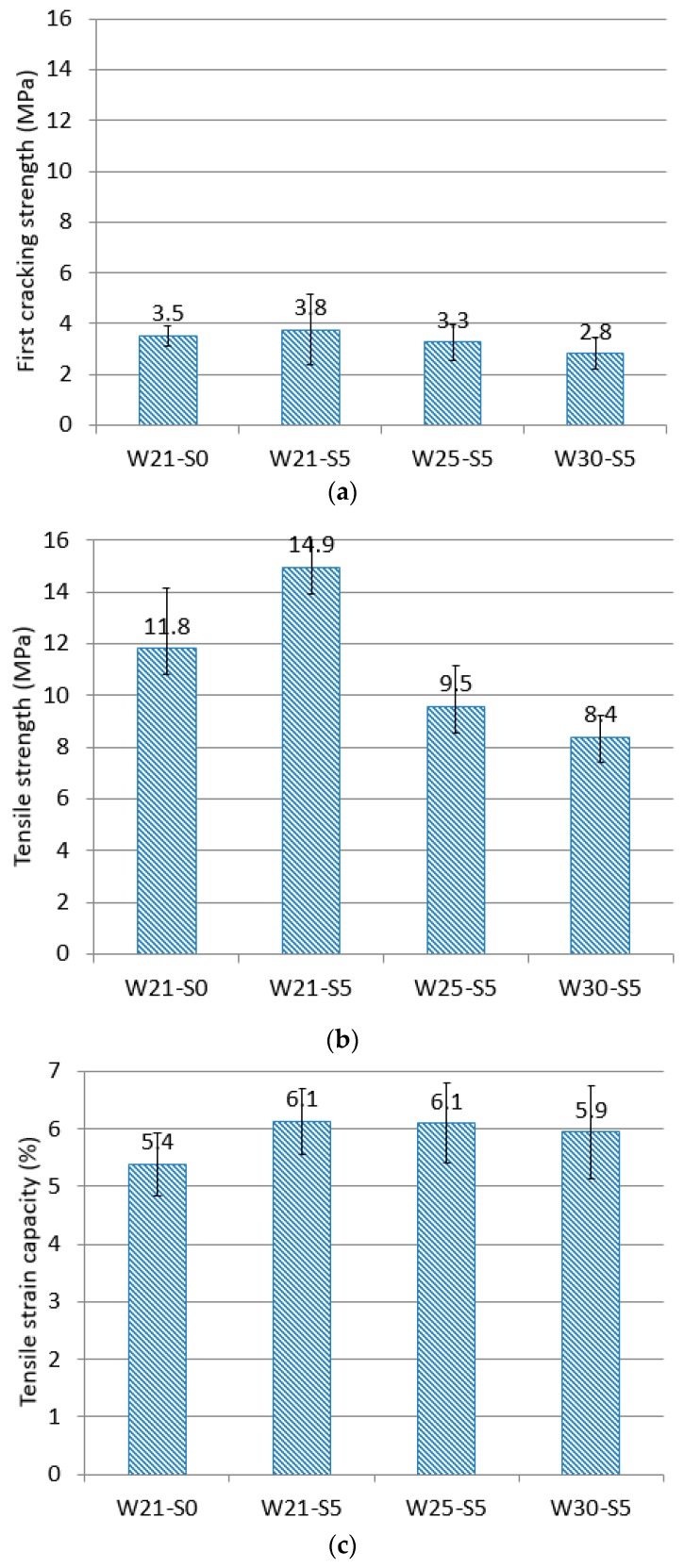
Tensile behavior of each mixture: (**a**) first cracking strength, (**b**) tensile strength, and (**c**) tensile strain capacity.

Figure 6 shows the first cracking strength, tensile strength, and tensile strain capacity, which represent the tensile performance of the individual mixtures. The first cracking strength means the stress measured at the generation of the first crack in the specimen, defined as the point on the tensile stress and strain curve where the stress is drastically changed [31]. Tensile strength refers to the maximum tensile stress. Tensile strain capacity is defined as the strain corresponding to the tensile strength.

The trend for first cracking strength was similar to the trend observed for compressive strength. However, unlike the results for compressive strength, the first cracking strength of the W30-S5 mixture was smaller than that of the W21-S0 mixture. In all the mixtures, the tensile strength was much higher than the first cracking strength, which means that the mixtures satisfied the strength condition, one of the conditions for multiple cracking [30]. In each mixture, the trend in tensile strength was similar to that of the first cracking strength. The tensile strength of the W21-S5 mixture was found to be 26.5% higher than that of the W21-S0 mixture. This increase was greater than the observed 18.8% increase in compressive strength. That is, the experimental results showed that incorporating ZSF had a greater effect on tensile strength than compressive strength. When the water-to-binder ratio was increased, the tensile strength decreased. The tensile strain capacity was high in all the mixtures. In fact, the tensile strain capacity of the high-ductile composites investigated in the present study was about two times higher on average than the tensile strain capacity (3%) of general high-ductile composites [32,33]. All of the mixtures prepared by incorporating ZSF showed a tensile strain capacity higher than that of the reference mixture. 

Overall, the W21-S5 mixture with 5% ZSF showed 18.8% higher compressive strength, 26.5% higher tensile strength, and 13.7% higher tensile strain capacity than the reference mixture. Achieving both high strength and high ductility in one material is highly desirable. The improved performance can benefit structures subjected to extreme loads, such as those from earthquakes and impact loads. These test results confirm that it is possible to improve not only the strength of fiber-reinforced alkali-activated slag-based composites, but also their ductility, by incorporating the proper amount of ZSF in the composite.

**Figure 7:**
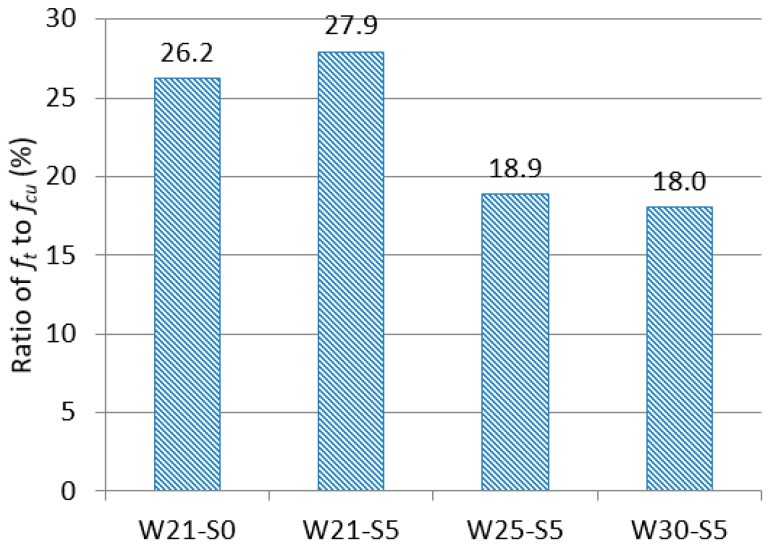
Ratio of tensile strength to compressive strength.

Figure 7 shows the ratio of tensile strength (*f_t_*) to compressive strength (*f_cu_*). In normal concrete, the ratio of tensile strength to compressive strength is approximately 10% [34]. By comparison, the ratio of tensile strength to compressive strength found in the composites investigated in the present study was very high. 

In ultimate strength design, the tensile strength of concrete is typically not considered, because the tensile strength is much smaller (about 10%) than the compressive strength. The tensile fracture strain at which cracks are generated (about 0.01%) is smaller than the compressive fracture strain rate (about 0.3%), and thus contributes little to load resistance. But a high tensile strength-to-compressive strength ratio means that the tensile behavior can be taken into consideration in the structural design. The tensile strength-to-compressive strength ratio of the mixtures investigated in the present study was more than two times higher than that of normal concrete, and the tensile strain capacity was several hundred times higher than that of normal concrete. Therefore, the mixtures investigated in the present study may contribute to improving the performance of structures.

**Figure 8:**
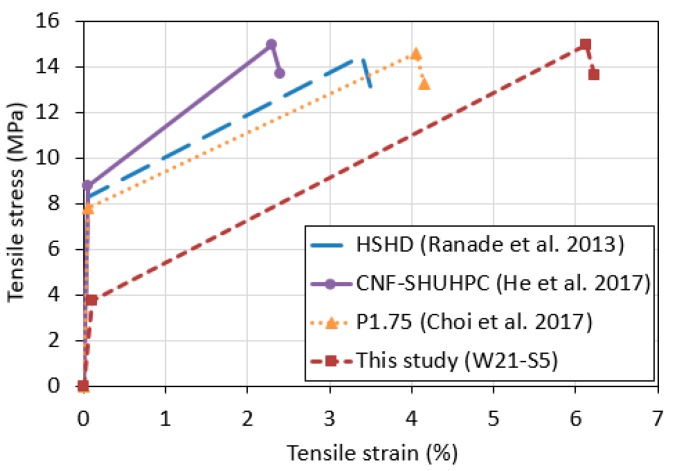
Comparison of tensile stress and strain curves of ultra-high performance concretes with high-ductility and the W21-S5 mixture.

Figure 8 compares the tensile stress and strain behavior of ultra-high performance concretes (UHPCs) with high-ductility, and the W21-S5 mixture prepared in the present study [30,35,36]. As shown in Figure 8, although the W21-S5 mixture showed a first cracking strength lower than those of the ductile UHPCs, it showed a similar tensile strength and excellent tensile strain capacity. 

Table 3 quantitatively compares their compressive strength and tensile performance. The compressive strength of the W21-S5 mixture was as low as 37% that of the ductile UHPCs, but the tensile strength was almost similar. Notably, while the tensile strength-to-compressive strength ratios of the ductile UHPCs was about 10%, that of the W21-S5 mixture was 27.6%, which is 2.34 to 3.16 times higher than the ductile UHPCs. The tensile strain capacity of the W21-S5 mixture was 1.5 to 2.7 times higher than those of the ductile UHPCs. 

Toughness, which indicates the capacity of a material to absorb externally applied energy until the material is broken, can be calculated from the tensile stress and the strain rate curve. The toughness of the W21-S5 mixture was 1.27 to 2.11 times higher than those of the ductile UHPCs. These results demonstrate the excellent tensile performance of the mixtures investigated in the present study, compared with the ductile UHPCs.

**Figure 9:**
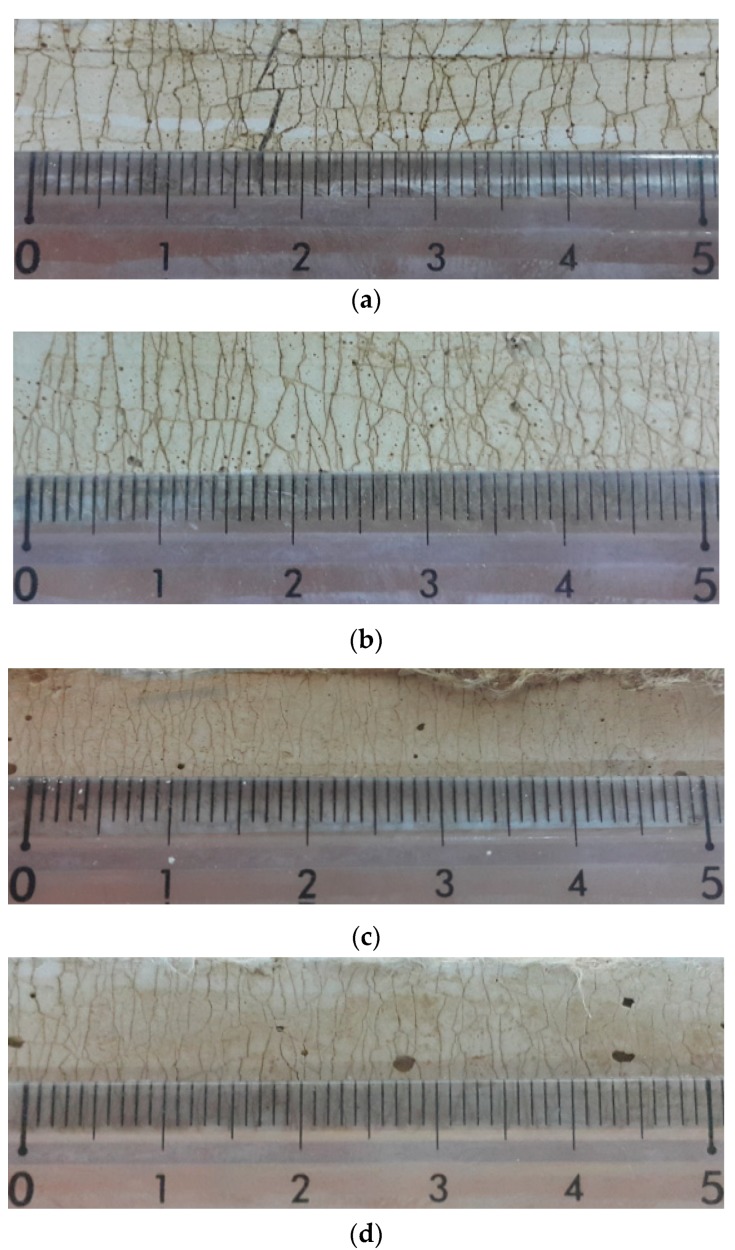
Representative cracking patterns: (**a**) W21-S0, (**b**) W21-S5, (**c**) W25-S5, and (**d**) W30-S5.

**Figure 10:**
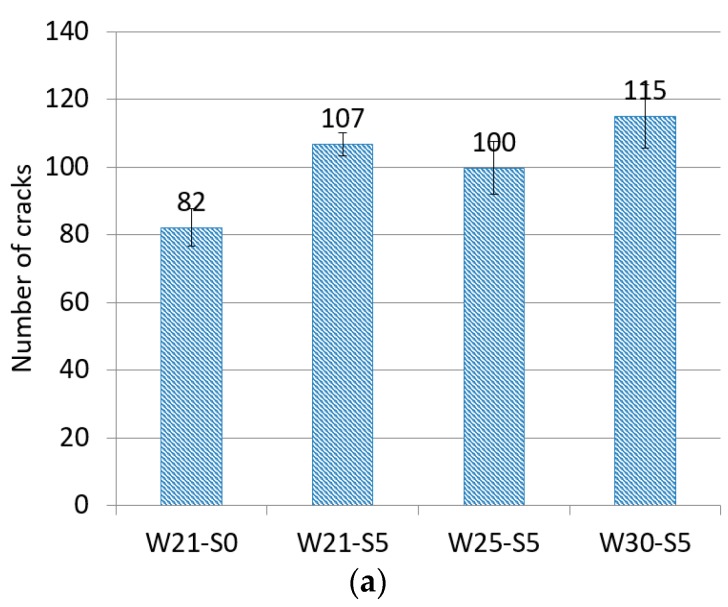

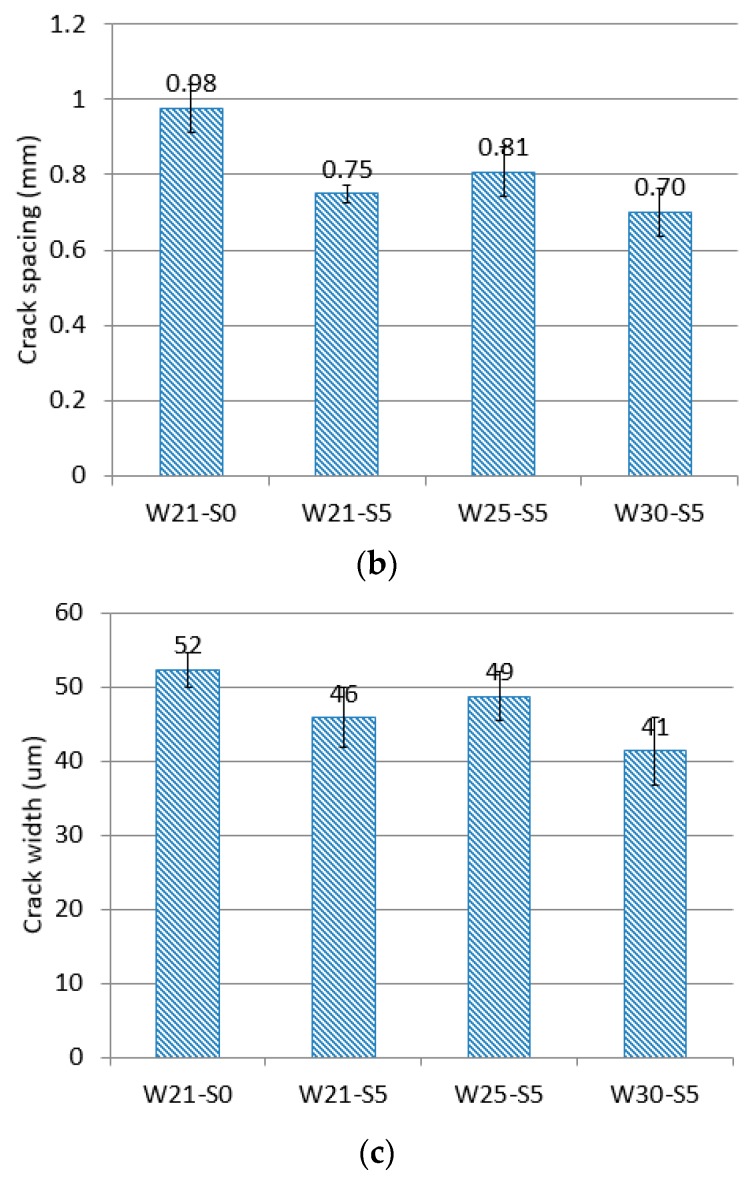
Average cracking patterns: (**a**) number of cracks within gauge length (80 mm), (**b**) crack spacing, and (**c**) crack width.

Figure 9 shows the cracking pattern of the mixtures investigated in the present study. Fine multiple cracks were generated in all the mixtures. Figure 10 shows the average number of cracks, the crack spacing, and crack width of the individual mixtures within the gauge length (80 mm), to quantitatively represent the cracking patterns.

It was observed that the number of cracks generated in the mixtures incorporating ZSF was about 30% greater than that of the reference mixture (W21-S0). It was found that the crack spacing and the crack width of the mixtures with ZSF were 23% and 13% smaller than that of reference mixture, respectively. In particular, compared to the reference mixture, the W21-S5 mixture, prepared by replacing 5% of the binding materials with ZSF, showed higher mechanical performance in terms of compressive strength, tensile strength, tensile strain capacity, and toughness, and better durability in terms of cracking patterns.

It is well known that crack width is closely related to the durability of materials and structures: the smaller the crack width, the higher the durability. As described above, the addition of ZSF might have increased the interfacial bond between the fiber and the matrix. When a crack occurs, the stress of the matrix at the crack’s surface is zero, and the external force is fully resisted by the bridging force of fibers at the cracks. As the distance from a crack is increased, the stress transferred from the fiber to the matrix is increased, thus increasing the stress applied to the matrix. A new crack is generated when the stress transferred from the fiber to the matrix is greater than the cracking strength of the matrix. At the same time, as the interfacial bond is increased, the distance between an existing crack and a newly generated crack is decreased [36]. 

Overall, it was determined that adding ZSF to the alkali-activated slag-based composite may improve not only mechanical performance but also durability.

**Figure 11:**
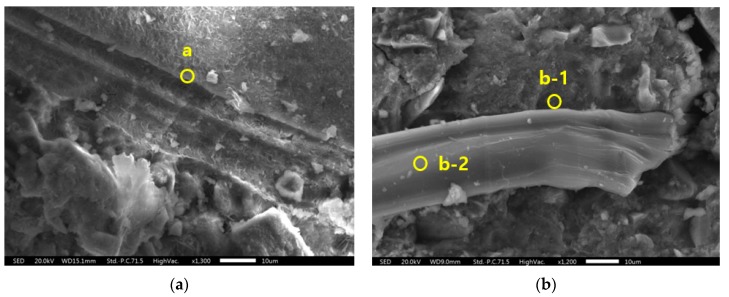
SEM images: (**a**) W21-S0 specimen and (**b**) W21-S5 specimen.

**Figure 12:**
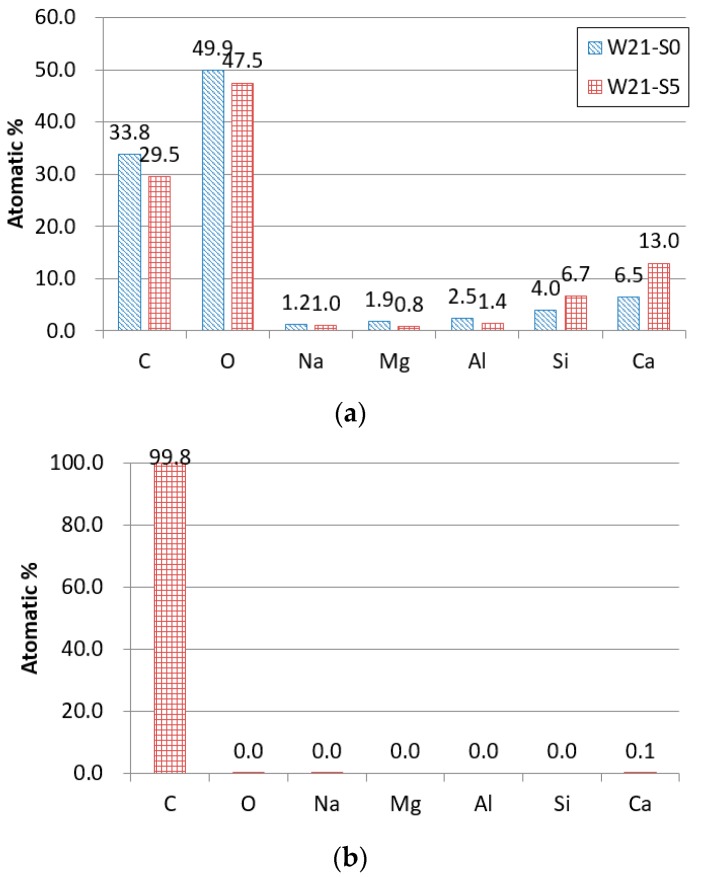
Chemical compositions: (**a**) positions a (W21-S0 specimen) and b-1 (W21-S5 specimen); (**b**) position b-2.

Figure 11 shows SEM images of the W21-S0 specimen and the W21-S5 specimen. As shown in Figure 11, there was no significant difference in the morphology and microstructures of the W21-S0 and the W21-S5 specimens. Next, the chemical compositions of each point shown in Figure 11 were analyzed by EDS, and the results are shown in Figure 12. Positions besides fibers were selected to identify the chemical composition of the matrix, and one position on the fiber was selected to identify the chemical composition of the fiber. A higher amount of silicate was observed in the W21-S5 specimen with added ZSF than the W21-S0 specimen. It seems that the observation of carbon at both positions is due to the fiber. The fiber was damaged during fiber pullout, which may have left a fiber component on the surface of the matrix. Most of the chemical composition of the fiber surface was carbon.

## 4. Conclusions

The present study was conducted to experimentally investigate the effect of added ZSF on the mechanical performance of fiber-reinforced alkali-activated slag-based composites. Experiments were performed to evaluate the density, compressive strength and tensile strength of the composite materials. From the experimental test results, it was determined that the strength and tensile strain capacity of a fiber-reinforced alkali-activated slag-based composite with a water-to-binder ratio of 21% can be improved by replacing 5% of the binding materials with ZSF. The compressive strength, tensile strength, and tensile strain capacity of the W21-S5 mixture, in which 5% of the binding materials was replaced with ZSF, were 18.8%, 26.5% and 13.7% higher than those of the reference mixture (W21-S0), respectively. The W21-S5 mixture exhibited a compressive strength of 53.5 MPa, a tensile strength of 14.9 MPa, and a tensile strain capacity of 6.1%. Adding ZSF to the fiber-reinforced alkali-activated slag-based composites increased the average number of cracks by 30%, and decreased the crack spacing and crack width by 23% and 13%, respectively. In particular, the W21-S5 mixture showed higher mechanical performance and better durability in terms of cracking patterns compared with the reference mixture (W21-S0). The compressive strength of the W21-S5 mixture was approximately 30% lower than that of the ductile UHPCs, but their tensile strength values were similar. While the tensile strength-to-compressive strength ratio of the ductile UHPCs was about 10%, that of the W21-S5 mixture was 27.6%, which was a maximum of 3.16 times higher than the ductile UHPC. The tensile strain capacity of the W21-S5 mixture was 1.5 to 2.7 times higher than the ductile UHPCs.

## Figures and Tables

**Table 1:** Chemical compositions of ground granulated blast furnace slag (GGBS) and zirconia silica fume (ZSF).

Materials	CaO	SiO_2_	Al_2_O_3_	MgO	SO_3_	TiO_2_	K_2_O	Fe_2_O_3_	MnO	ZrO_2_	Etc.
GGBS	40.4	30.6	13.8	8.0	4.0	0.9	0.5	0.5	0.5	-	0.8
Zirconia silica fume	0.02	94.51	1.37	0.09	-	0.04	0.02	0.51	0.01	1.51	1.92

**Table 2:** Mix proportions.

Mixture	Binder				Water	HRWRA	Defoamer	Fiber (vol.%)
	GGBS	Ca(OH)_2_	Na_2_SO_4_	ZSF				
W21-S0	0.895	0.075	0.03	-	0.21	0.025	0.001	2.0
W21-S5	0.85	0.071	0.029	0.05	0.21	0.030	0.001	2.0
W25-S5	0.85	0.071	0.029	0.05	0.25	0.014	0.001	2.0
W30-S5	0.85	0.071	0.029	0.05	0.30	0.007	0.001	2.0

Note: Mass ratios of binding material weight, except fiber.

**Table 3:** Comparison of performance of ultra-high performance concretes with high-ductility and the W21-S5 mixture.

Composite	Compressive Strength (MPa)	Tensile Strength (MPa)	Ratio of *f_t_* to *f_cu_* (%)	Tensile Strain Capacity (%)	Toughness (MPa m/m)
Ranade et al. 2013	166	14.5	8.7	3.4	0.38
He et al. 2017	153	15.0	9.8	2.3	0.27
Choi et al. 2017	124	14.6	11.8	4.1	0.45
This study (W21-S5)	54	14.9	27.6	6.1	0.57

## References

[1] Choi M.S., Kang S.-T., Lee B.Y., Koh K.-T., Ryu G.-S. (2016). Improvement in predicting the post-cracking tensile behavior of ultra-high performance cementitious composites based on fiber orientation distribution. Materials.

[2] Smarzewski P., Barnat-Hunek D. (2017). Effect of fiber hybridization on durability related properties of ultra-high performance concrete. Int. J. Concr. Struct. Mater..

[3] Malhotra V. (2002). Introduction: Sustainable development and concrete technology. Concr. Int..

[4] Damtoft J., Lukasik J., Herfort D., Sorrentino D., Gartner E. (2008). Sustainable development and climate change initiatives. Cem. Concr. Res..

[5] Choi J.-I., Lee B.Y., Ranade R., Li V.C., Lee Y. (2016). Ultra-high-ductile behavior of a polyethylene fiber-reinforced alkali-activated slag-based composite. Cem. Concr. Compos..

[6] Choi J.-I., Song K.-I., Song J.-K., Lee B.Y. (2016). Composite properties of high-strength polyethylene fiber-reinforced cement and cementless composites. Compos. Struct..

[7] Choi S.-J., Choi J.-I., Song J.-K., Lee B.Y. (2015). Rheological and mechanical properties of fiber-reinforced alkali-activated composite. Constr. Build. Mater..

[8] Lee B.Y., Cho C.-G., Lim H.-J., Song J.-K., Yang K.-H., Li V.C. (2012). Strain hardening fiber reinforced alkali-activated mortar—a feasibility study. Constr. Build. Mater..

[9] Lee Y., Choi J.-I., Kim H.-K., Lee B.Y. (2017). Effects of a defoamer on the compressive strength and tensile behavior of alkali-activated slag-based cementless composite reinforced by polyethylene fiber. Compos. Struct..

[10] Nematollahi B., Sanjayan J., Ahmed Shaikh F.U. (2015). Tensile strain hardening behavior of PVA fiber-reinforced engineered geopolymer composite. J. Mater. Civ. Eng..

[11] Ohno M., Li V.C. (2014). A feasibility study of strain hardening fiber reinforced fly ash-based geopolymer composites. Constr. Build. Mater..

[12] Kwon S.-J., Choi J.-I., Nguyen H.H., Lee B.Y. (2018). Tensile strain-hardening behaviors and crack patterns of slag-based fiber-reinforced composites. Comput. Concr..

[13] Nguyễn H.H., Choi J.-I., Kim H.-K., Lee B.Y. (2019). Effects of the type of activator on the self-healing ability of fiber-reinforced alkali-activated slag-based composites at an early age. Constr. Build. Mater..

[14] Nguyễn H.H., Choi J.-I., Song K.-I., Song J.-K., Huh J., Lee B.Y. (2018). Self-healing properties of cement-based and alkali-activated slag-based fiber-reinforced composites. Constr. Build. Mater..

[15] Oakes L., Magee B., Mcllhagger A., McCartney M. (2019). Strength prediction and mix design procedures for geopolymer and alkali-activated cement mortars comprising a wide range of environmentally responsible binder systems. J. Struct. Integrity Maint..

[16] Ohno M., Li V.C. (2018). An integrated design method of engineered geopolymer composite. Cem. Concr. Compos..

[17] Shaikh F.U.A., Fairchild A., Zammar R. (2018). Comparative strain and deflection hardening behaviour of polyethylene fibre reinforced ambient air and heat cured geopolymer composites. Constr. Build. Mater..

[18] De Domenico D., Faleschini F., Pellegrino C., Ricciardi G. (2018). Structural behavior of RC beams containing EAF slag as recycled aggregate: Numerical versus experimental results. Constr. Build. Mater..

[19] Faleschini F., Pellegrino C. (2013). Experimental behavior of reinforced concrete beams with electric arc furnace slag as recycled aggregate. ACI Mater. J..

[20] Rondi L., Bregoli G., Sorlini S., Cominoli L., Collivignarelli C., Plizzari G. (2016). Concrete with EAF steel slag as aggregate: A comprehensive technical and environmental characterisation. Compos. Part B.

[21] Bhanja S., Sengupta B. (2005). Influence of silica fume on the tensile strength of concrete. Cem. Concr. Res..

[22] Li Z., Wang L., Ma G. (2018). Method for the enhancement of buildability and bending resistance of 3D printable tailing mortar. Int. J. Concr. Struct. Mater..

[23] Long G., Wang X., Xie Y. (2002). Very-high-performance concrete with ultrafine powders. Cem. Concr. Res..

[24] Aıtcin P. (2003). The durability characteristics of high performance concrete: A review. Cem. Concr. Compos..

[25] JSCE (2008). Recommendations for Design and Construction of High Performance Fiber Reinforced Cement Composites with Multiple Fine Cracks (HPFRCC).

[26] ASTM (2013). Standard Test Method for Density, Absorption, and Voids in Hardened Concrete.

[27] ASTM (2007). Standard Test Method for Compressive Strength of Hydraulic Cement Mortars (Using 2-in. Or [50-mm] Cube Specimens).

[28] Bischoff P.H., Perry S. (1991). Compressive behaviour of concrete at high strain rates. Mater. Struct..

[29] Hentz S., Donzé F.V., Daudeville L. (2004). Discrete element modelling of concrete submitted to dynamic loading at high strain rates. Comput. Struct..

[30] Ranade R., Li V.C., Stults M.D., Heard W.F., Rushing T.S. (2013). Composite properties of high-strength, high-ductility concrete. ACI Mater. J..

[31] Li V.C. (1993). From micromechanics to structural engineering-the design of cementitous composites for civil engineering applications. J. Struct. Mech. Earthquake Eng..

[32] Li V.C. (2012). Tailoring ECC for special attributes: A review. Int. J. Concr. Struct. Mater..

[33] Xu S.-L., Cai X.-R. (2010). Experimental study and theoretical models on compressive properties of ultrahigh toughness cementitious composites. J. Mater. Civ. Eng..

[34] Mindess S., Young J.F., Darwin D. (2002). Concrete.

[35] Choi J.-I., Jang S.Y., Kwon S.-J., Lee B.Y. (2017). Tensile behavior and cracking pattern of an ultra-high performance mortar reinforced by polyethylene fiber. Adv. Mater. Sci. Eng..

[36] He S., Qiu J., Li J., Yang E.-H. (2017). Strain hardening ultra-high performance concrete (SHUHPC) incorporating CNF-coated polyethylene fibers. Cem. Concr. Res..

